# A qualitative study on the experiences of family caregivers of children with End Stage Kidney Disease (ESKD)

**DOI:** 10.1186/s13030-024-00314-8

**Published:** 2024-08-16

**Authors:** Edward Appiah Boateng, Mabel Baaba Bisiw, Rosemary Agyapomah, Isaac Enyemadze, Joana Kyei-Dompim, Samuel Peprah Kumi, Dorothy Serwaa Boakye

**Affiliations:** 1 Department of Nursing, School of Nursing and Midwifery, KNUST, Kumasi, Ghana; 2 Nursing and Midwifery Training College, Kokofu, Ghana; 3 Kumasi High School, Kumasi, Ghana; 4 Department of Midwifery, School of Nursing and Midwifery, KNUST, Kumasi, Ghana; 5 Nurses’ Training College, Sampa, Ghana; 6 Department of Health Administration and Education, University of Education, Winneba, Ghana

**Keywords:** End stage kidney disease, Children, Experiences, Family caregivers, Phenomenology, Qualitative study

## Abstract

**Background:**

Family caregivers, mostly parents, are greatly involved in the care of their children with end stage kidney disease (ESKD) globally. Yet, the experiences of these caregivers and the demands placed on them by the caregiving role have not been explored or documented in Ghana. This study explored how caregiving affects the psychological, physical, social, and spiritual well-being of family caregivers of children with end stage kidney disease (ESKD) in Ghana.

**Methods:**

A phenomenological approach with the purposive sampling technique was used to gather data from 12 family caregivers of children with ESKD at a pediatric renal unit in Ghana. A semi-structured interview guide was constructed based on the constructs of the City of Hope Quality of Life (QoL) Family Caregiver Model and the research objectives. Colaizzi’s thematic analysis approach was utilized to analyze data for this study. Themes were organized under the domains of the chosen model, and a new theme outside these domains was also generated.

**Results:**

The majority of the family caregivers experienced anxiety, fear, uncertainty, and hopelessness in response to the children’s diagnosis and care. The thought of the possibility of the children dying was deeply traumatizing for our participants. Most participants reported bodily pains and physical ailments because of lifting and caring for the children. Financial constraint was also a key issue for all the family caregivers. Most of them received diverse support from their families and loved ones. Due to the demanding nature of the care, most family caregivers had to change or quit their jobs. They coped with the challenges through prayers, participating in religious activities, and being hopeful in God for healing.

**Conclusion:**

All the family caregivers had their psychological well-being compromised as a result of the challenges they encountered physically, socially, and spiritually. Continuous psychosocial support, funding support, and review of policies on leave for civil workers with children diagnosed with ESKD are urgently required.

**Supplementary Information:**

The online version contains supplementary material available at 10.1186/s13030-024-00314-8.

## Background

End stage kidney disease (ESKD), the last of the five stages of chronic kidney disease (Table 1), has devastating effects on both the patient and the family caregiver. Yet, family caregivers have received little attention in the literature [1–3]. The care provided by family caregivers to individuals living with ESKD is significantly associated with successful adherence to treatment, thereby improving their survival rate and quality of life [4, 5]. Hence, family caregivers must have adequate knowledge, skills, and psychosocial and physical strength to provide care to their relatives and to cope with the demands of care without endangering their well-being [6].

**Table 1:** GFR categories in CKD [7]

GFR Category	GFR (ml/min per1.73m^2^)	Terms
G1	≥ 90	Normal or high
G2	60–89	Mildly decreased^a^
G3a	45–59	Mildly to moderately decreased
G3b	30–44	Moderately to severely decreased
G4	15–29	Severely decreased
G5	< 15	Kidney failure

CKD: chronic kidney disease; GFR: glomerular filtration rate; ^a^Relative to the young adult level. In the absence of evidence of kidney damage, neither G1 nor G2 fulfills the criteria for CKD

The provision of care to children with ESKD particularly comes with many laborious and intensive activities, including the management of symptoms and complications which are highly distressing for the family caregiver, mostly the parents, and reduce their quality of life [8, 9]. Key problems experienced by parents of children with ESKD include psychological and emotional problems, those related to the treatment of the child, support needs, and impact on their personal, financial, social, and family life [8, 10, 11]. The struggles these caregivers face, including the stressful hospital environment, partaking in and implementing decisions regarding complex medical interventions, and maintaining family norms and dynamics have been well documented [12]. Indeed, the chronic nature of the disease, in addition to frequent hospitalizations makes it extremely difficult for caregivers of children with CKD to adapt to the caregiving role as each stage of the disease and its respective treatment strategies create different concerns for them [12, 13]. Their parenting experience becomes medicalized, requiring them to find a balance between their roles as parents while providing sometimes complex technological medical interventions to their affected child [12].

Hopelessness and despair have also been reported as well as the lack of an opportunity to prepare for the changes that come with the disease affecting their child and their evolving roles [8]. The fear of losing the child and uncertainties for the future also mostly burden these caregivers [9]. They find these experiences extremely distressing and spend much time worrying about whether they can cope with the expectations ‘imposed’ on them by the prevailing circumstances [12]. Interestingly, parents whose experiences are reported in studies are mostly mothers, and various reasons, including busyness, have been cited for the lack of involvement of fathers in the primary caregiver role [9, 12–14]. The implication is that the quality of life of mothers of children with ESKD is severely affected, as compared to the fathers [15].

The literature on ESKD in Ghana has mainly focused on adult patients and their family caregivers [16–20]. Studies that have focused on the experiences of parents of children with ESKD have largely been conducted outside Ghana [8–10, 12, 13, 15]. The only study involving caregivers of children with CKD in Ghana focused on their perspectives on factors that caused delays in seeking medical care for the children, not on their caregiving experiences [21]. Understanding caregivers’ experiences will broaden the perspectives of healthcare providers to enable them to recommend effective coping strategies and support while caring for their children with ESKD [10]. This study thus explored the physical, psychological, spiritual, and social experiences of parents of children with ESKD using the City of Hope Quality of Life (QoL) Family Caregiver Model [22] as a guide.

## Methods

### Study setting

The study was conducted at the pediatric renal unit of a teaching hospital in Ghana. The unit had three specialist nephrology nurses and three other nurses trained on the job at the time of data collection. The unit only offers peritoneal dialysis for children with acute kidney injury and conservative treatment for children with ESKD up to the age of 14 years – there were plans to commence hemodialysis services for children at the time of the study. Only children who were ‘big’ enough to allow for adult catheters to be used and whose families had the financial means to pay received hemodialysis in the adult hemodialysis unit. Children with ESKD visit the clinic based on their response to treatment and their proximity to the hospital. Those who live far from the hospital and are stable visit quarterly. At the same time, those who need close attention are reviewed monthly. While the National Health Insurance Scheme (NHIS) in Ghana aims to promote universal access to healthcare, the scheme does not cover the cost of treatment for certain diseases, including ESKD. Thus, individuals with ESKD and their significant others have to make out-of-pocket payments for treatment and key diagnostic examinations, putting a huge financial burden on them [16, 17, 19].

### Study design

This study employed a phenomenological approach since much is not known about the experiences of family caregivers of children with ESKD in Ghana.

### Research participants and sampling

The study comprised family caregivers of children with ESKD receiving treatment at the teaching hospital. While this was mainly expected to be their biological parents, the study was also open to any other adult who provided informal care for the child with ESKD. Indeed, in Ghana, social parenthood where adults other than biological parents provide care for children is a common practice, although this seems to be weakening [23, 24]. The purposive sampling technique was used to recruit 12 family caregivers for the study. The sample size was informed by data saturation where no new themes were derived from the data in line with the objectives of the study as well as the timelines for the completion of the study [25]. Two interviews were conducted to pretest the interview guide that was developed for this study, after which minor revisions were made to clarify some questions to elicit in-depth responses from participants.

### Research team

The team comprised experienced researchers as well as graduate students pursuing MPhil Nursing at KNUST at the time of the study. All members of the team are fluent in English and Twi (the most common Ghanaian language in the setting of the study). While the lead researcher (EAB) has done some work on ESKD in Ghana, all other authors brought their diverse views to the fore during the conceptualization of the study as well as discussions on the data analysis process.

### Data collection

After obtaining ethics approval, EAB and MBB visited the pediatric renal unit and explained the purpose, objectives, and process of data collection to the head of the unit. Data collection was led by MBB who interacted one-on-one with prospective participants to explain the objectives of the study and invited them to participate. All those who were approached agreed to be part of the study. A semi-structured interview guide was developed after reviewing relevant literature. A consent form was signed by each participant before being interviewed. Individual face-to-face interviews were conducted in Twi or English, based on each participant’s choice. Each interview session lasted between 25 and 40 min. The interview was initiated with the question: “Please tell me how you felt after you were informed of your child’s diagnosis”. Probes such as “Kindly elaborate further” or “What do you mean”? were introduced to assist participants in providing in-depth descriptions of their experiences. All interviews were conducted at a place within the hospital that ensured privacy during the session – mainly in a consulting room. No third parties were allowed into the interview premises. The interviews were audio recorded with the consent of participants. Data collection lasted for 4 weeks. There were no repeat interviews. The interviews done in the local dialect were transcribed verbatim, and then translated into English for analysis. Field notes were taken to record observations and mannerisms that were not captured through the audio recording during data collection.

### Data management and analysis

Data collection and analysis were conducted simultaneously. The interview recordings were transcribed verbatim at the end of each session. The audio recordings and transcripts from the study were kept on a password-protected computer. To prevent data loss, backups on a password-protected external hard drive and flash drive were made [26]. The Colaizzi [27] thematic analysis strategy guided data analysis. The City of Hope Quality of Life (QoL) Family Caregiver Model [22] proposes 4 key concepts that were adopted in the design of the instrument for data collection and applied as a lens during data analysis to identify patterns in the participants’ narratives. The QoL model was a good fit for this study because it examines how caregiving interferes with the physical, psychological, social, and spiritual well-being of caregivers.

The pre-existing themes from the adopted model guided the data analysis. Data analysis was led by MBB, with significant inputs from all authors during regular discussions on the study. The transcripts were read several times, key and significant statements were noted and extracted, and meanings were formulated for the significant statements. Similar phrases were then grouped to reflect a unique structure of cluster of themes using the QoL Family Caregiver Model. The grouped clusters of themes that reflected significant concepts were merged to form a unique theme [28–30]. The descriptions of the meanings were examined, and the findings were then validated by nine of the participants. Table 2 provides a summary of the data analysis process.

**Table 2:** Colaizzi’s steps as employed in the data analysis process

Colaizzi’s Steps	Application in the Study
1. Reading and re-reading the transcript	Transcripts were read several times to ensure familiarity with the content.
2. Extracting significant statements	Ideas, thoughts and feelings that disclosed participant experiences in line with our study objectives were highlighted and extracted.
3. Formulating meanings from significant statements	Similar phrases were noted and recorded, noting their lines and pages. These identified similar phrases helped to group, retrieve, organize, and analyze the data. Meanings were formulated from the significant statements. All researchers had frequent discussions on the formulated meanings and compared them with the data to ensure consistency.
4. Clustering formulated meanings into themes	Upon reaching a consensus on the formulated meanings, similar phrases were grouped to reflect a unique structure of cluster of themes using the QoL model applied to family caregivers. Grouped clusters of themes that reflected significant concepts were merged to form a theme, according to the constructs of the QoL model applied to family caregivers.
5. Developing an exhaustive description of the phenomenon based on themes	The meanings of the themes were studied and then integrated into comprehensive descriptions of the lived experiences of family caregivers of children with ESKD.
6. Description of fundamental structure of the phenomenon	The descriptions of the meanings of experiences of family caregivers of children with ESKD were examined and formulated into concise statements based on the exhaustive descriptions. Frequent discussions were held among all researchers until a consensus was reached that the statements truly reflected the experiences of family caregivers of children with ESKD.
7. Verification of the fundamental structure.	Validation of the findings was sought from the study participants. Nine participants were contacted with the findings and all confirmed that the interpretations of the findings were true reflections of their experiences.

### Trustworthiness

Trustworthiness was established through the four main criteria proposed by Lincoln and Guba [31]. To ensure credibility and confirmability, only participants who met the inclusion criteria were recruited and member checking was also achieved by nine participants confirming that the findings of the study were true reflections of their descriptions [29, 31, 32]. A detailed description of all the methodological processes employed in this study and the context of the study have been provided, which enhances the dependability and transferability of the study.

## Results

Five themes were generated from the analysis of data for this study, four in line with the City of Hope Quality of Life Family Caregiver Model and an additional theme titled ‘health education as a coping strategy’. The demographic characteristics of the participants and the themes are presented below.

### Demographic characteristics of participants

A total of 12 family caregivers, comprising eleven women and one man, were recruited for the study. Their ages ranged from 26 to 69 years. The youngest child with ESKD in this study was 5 years old while the oldest was 13 years old. The participants had provided care for the children for at least one year. All the participants were Christians. Three participants were fully employed, six were self-employed and three were unemployed. Table 3 summarizes the demographic data of the participants.

**Table 3:** Demographic data of the study participants

Participant	Age Range (years)	Employment status	Relationship to child	Age of child (years)	Duration of care (years)
P1	26–35	Unemployed	Mother	6	3–4
P2	26–35	Self-employed	Mother	7	1–2
P3	36–45	Full employment	Mother	13	3–4
P4	56–65	Unemployed	Grandmother	6	1–2
P5	36–45	Self-employed	Aunt	12	3–4
P6	26–35	Self-employed	Mother	9	1–2
P7	26–35	Full employment	Mother	10	3–4
P8	36–45	Unemployed	Mother	13	7–8
P9	26–35	Self-employed	Sister	9	3–4
P10	36–45	Full employment	Father	9	5–6
P11	46–55	Self-employed	Grandmother	5	1–2
P12	36–45	Self-employed	Mother	13	5–6

### Psychological well-being of family caregivers of children with ESKD

The family caregivers experienced anxiety, depression, surprise, anger, and hopelessness while providing care for the children with ESKD. Participants generally observed changes in the children before the diagnosis. Their awareness of these changes made them seek medical attention. The most common observation was generalized edema.

*“She became so swelled up that more than two people had to lift her to urinate or perform other activities of daily living”.***P9**.

The generalized edema, unexpected diagnosis of ESKD, learning of the possible fatality associated with the condition, and financial challenges made most of the family caregivers anxious. The attribution of the disease to curses by some members of the community and the fear of the children dying increased their anxiety.

*“I was downhearted because I felt like I might not be able to provide all that he needs to survive*,* and I will eventually lose him.”***P8**.

*“I was disheartened*,* and I cried a lot because our co-tenants thought that my child had been cursed; others also came to our house to ask several questions.”***P7**.

Participants generally reported being shocked, angry, and surprised upon hearing the diagnosis of their children and this made it difficult for them to accept it. Some thought that ESKD only affected adults, not children, and this made it extra difficult for them to come to terms with the diagnosis. They believed that they had taken good care of their children and did not understand where their parenting fell short, which they felt was related to the diagnosis of ESKD.

*“I was angry in my heart upon hearing the diagnosis because when I compare my child with that of my friends*,* especially when I know I took good care of him*,* and for my child to end up with this disease; I became furious”.***P10**.

The inability to afford expensive medications, lack of improvement in the condition of the child, and their inability to do anything about the situation made the family caregivers hopeless.

*“I lost all hope because when I looked at my child*,* I knew there was nothing I could do and that I would not be able to take him back home with me.”***P7**.

### Physical well-being of family caregivers of children with ESKD

The family caregivers experienced pain and physical ailment, impaired sleep, stress, loss of appetite, weight loss, and poor grooming which were attributed to the caregiving role. They experienced pain from lifting and assisting the children to perform activities of daily living. A participant suffered a knee injury from a fall while on the way to the hospital. Some experienced dizziness or developed hypertension.

*“It was not easy for me at all; I felt a lot of pain in my body*,* thighs*,* and the lower part of my buttocks from lifting and helping her out of bed.”***P9**.

*“It is very difficult for me to eat*,* because if I put the food into my mouth and I remember or see her state then I lose appetite instantly.”***P4**.

Some participants reported an inability to either rest or sleep, while others felt exhausted and stressed out. They could not sleep because they felt the children were uncomfortable and they had to stay awake and watch over them. Some could not sleep because they were anxious about the condition of their children.

*“I could not sleep*,* I had to stay awake and observe or touch him to see how he was fairing.”***P11**.

Adhering to hospital appointments and reviews, and pressure from work competing with the care of the children was stressful and distressing and made some participants consider taking the child for alternative treatment and support.

*“I am stressed out*,* at a point in time I wanted to stop bringing him for his follow-up appointments and go back to my hometown to receive help in his care regardless of the consequences; there is a lot of pressure on me also from my workplace in addition to caring for my son.”***P8**.

Others also reported neglecting their hygiene, keeping their hair unkempt, dressing shabbily, and not buying new clothing, suggesting that their lives had seemingly come to a standstill following the diagnosis. Some female participants had to change their hairstyle, to avoid having to go to the salon often, to save time and money for the care of their children.

*“My hair does not look attractive; for 4 years now; I do not even feel the essence of going to the salon to treat my hair*,* so I have cut down all my hair. Also*,* since he got sick*,* I have never bought anything new for myself*,* personally*,* all the things I have are my old clothing…it is like I am not bothered about my looks and my skin. It is not something I even feel like doing*,* I am waiting for him to recover.”***P12**.

### Social well-being of family caregivers of children with ESKD

The family caregivers experienced some challenges in their relationships with their spouses, family members, and friends. While others experienced distortions in their family roles and responsibilities, leading to role conflicts in some homes, others enjoyed family support.

*“My ex-husband and I were not in a good relationship*,* but our relationship became better when our child got sick.”***P7**.

Most participants received diverse support, however. The support came from healthcare workers, family members, coworkers, church, co-tenants, and friends. The support was in the form of health education, financial assistance, prayers, taking up roles, and being given time off at the workplace. Participants considered the support as the reason for the children’s continual existence. However, some participants did not receive such support, making the caregiving role more distressing for them.

*“My mum was of great help as she took care of her while on admission*,* so I was able to work and get money to support her care.”***P2**.

All the participants expressed that the cost of treatment and care was very high. The financial burden led to anxiety, stress, and depression. Some could not meet the financial demands of the treatment for ESKD which led to a loss of concentration at work; others could not combine the care and their work, so they had to resign. Others also had issues with punctuality at work and were forced to apply for leave without pay to enable them to care for the children.

*“There wasn’t anyone to take care of him except me*,* so I had to stop working to take care of him in the hospital.”****P3***.

### Spiritual well-being of family caregivers of children with ESKD

The family caregivers related the meaning of the children’s illness to several factors, including curses, natural occurrences, and adult disease. Some felt that the disease was a consequence of their transgressions in the past while others felt it was a test of their faith. While some depended on God for the healing of the children by visiting several churches, others questioned God because they could not comprehend why such a disease could affect a child.

*“I have been to several churches for prayers for my child’s healing and well-being.”***P4**.

“*When we hear of kidney disease*,* we know it affects adults who drink and smoke*,* but not children.”***P2**.

Some of the participants strengthened their relationship with God by praying more and attending church services more regularly than previously. Prayers helped some to cope with the situation:

*“I added prayers to everything I did and that has helped me to cope and carry us this far – the reason he is still alive today.”***P12**.

### Health education as a coping strategy

This is a new theme that emerged from the study. It describes the positive informational support that enabled the participants to provide care for the children. They disclosed that health education received from the healthcare workers enabled them to monitor the children at home, watch out for warning signs, and adhere to dietary restrictions.

*“We were taught on how to take care of the children*,* especially by drawing them closer*,* what food should be given and not given*,* things to watch out for and report at the hospital.”****P1***.

In addition, health education helped the majority of the participants to adhere to the scheduled hospital appointments and treatment regimen for their children, and this resulted in positive prognostic outcomes for them.

*“As I kept coming for the follow-up appointments as scheduled*,* I noticed a lot of positive changes in my child.”****P4***.

In summary, participants described experiences that affected their psychological, physical, social, and spiritual well-being. Health education was identified as a tool that has been very helpful in empowering participants to provide care for their children while hoping for a good prognosis.

## Discussion

This study explored the experiences of family caregivers of children with ESKD and is the first of its kind in Ghana. It reports that the caregiving role affects the psychological, physical, social, and spiritual well-being of the family caregivers of children with ESKD.

Anxiety, fear, uncertainty, depression, and hopelessness were the diverse psychological experiences among our participants. Anxiety is common among family caregivers of children with ESKD [8, 33–35] and different reasons have been attributed to this. Tong, Lowe [12] attributed the anxiety of the family caregivers to a total loss of control over the health of their children, while Alnazly and Samara [33], Rodrigues de Lima, Flôres Cosentino [34], Salehitali, Ahmadi [35] attributed anxiety to the worsening of the children’s condition. Our participants mainly explained that they were anxious because they initially believed that ESKD affects only adults, is possibly fatal, financially draining, and were uncertain about the prognosis. The changes that are observed in the child with ESKD also cause worries and fear among family caregivers as they cause several limitations in the child (Wightman et al., 2019). Depression has also been identified as a major psychological issue among family caregivers of children with ESKD [12, 36–40]. Our participants reported being depressed, attributing it to the burden of caregiving, loss of social life, and the time, energy, and attention channeled into the care of their children, feeling that ESKD had taken everything from them. Some lost hope because they felt they could not do much about the child’s condition and feared losing them [9]. Similar sentiments have also been shared by family caregivers of children with cancer in Ghana [41, 42], suggesting that caring for a child with a chronic and potentially terminal disease arouses similar experiences in Ghana and other parts of the world.

Family caregivers of children with ESKD continuously have their physical well-being negatively impacted [6, 43]. The experience of pain and physical ailment is recognized in several studies [33, 34]. Our participants experienced pain in various parts of their bodies, attributable to lifting and assisting the children in performing activities of daily living. High blood pressure and general deterioration of health are also common findings among family caregivers [33] as well as neglect of personal grooming when caring for children with ESKD [44]. Impaired rest and sleep patterns were also commonly reported among our participants, attributable to anxiety and the need to stay awake to cater to the needs of their child [1, 33, 35, 37, 45].

The social well-being of family caregivers of children with ESKD is also affected, sometimes even affecting relationship between parents. This is mostly because the demands of care prevent them from making time for social activities [10]. Interestingly, one of our participants noted that her relationship with her ex-husband had improved as they needed to work closely together to cater to the needs of their child with ESKD. Those who continually receive social support from family members, health professionals, church, and significant others find this comforting and cope well with the stresses associated with the care [34, 46]. Notwithstanding the support received, financial challenges compelled many participants to sell their properties, postpone projects, and avoid shopping to save enough money for the treatment, which is commonplace in low- and middle-income countries [1, 47, 48]. The financial challenges are compounded by the loss of jobs, resignation, or prolonged absence without any form of compensation due to the care, and our participants were not exempted [10, 45, 49].

Among the many causes that our participants described for ESKD is curses. The perceived relationship between ESKD and spiritual causes is commonplace in Ghana and other similar settings [16]. Usually, questions are asked as individuals do a thorough mental search to identify whether they have committed a transgression that may have resulted in the development of the disease. Some of our participants felt that their children were being punished for their parents’ wrongdoings or that their faith in God was being tested. Some family caregivers feel guilty and blame themselves for somehow contributing to the occurrence of the disease [50]. Such an association leads to the pursuant of spiritual ideologies when managing the condition. This usually creates an air of doubt even after the diagnosis has been confirmed and treatment initiated and sometimes affects their practices [10, 16, 51]. This made faith in God a major avenue for coping with the informal caregiving role among our participants, once they believed that spirituality is key to the development as well as treatment of the disease. Some hoped that their belief in God could bring some relief to their wards, and this resulted in the development of some positive attitudes toward managing the disease. Their previous experiences in life gave them the serenity of mind to provide care without much guilt while improving their self-esteem [52, 53]. Prayers are also considered a major coping strategy for the feelings of guilt and the day-to-day challenges experienced by family caregivers of children with ESKD [33, 45].

Health education had a significant impact on the caring roles of family caregivers. Previous studies have identified informational support as an unmet need of family caregivers of children with ESKD [34, 53]. A recent review on knowledge requirements and unmet needs of family caregivers of patients with end-stage kidney disease also highlighted the difficulties family caregivers experience with obtaining information from health workers [54]. However, family caregivers in our study reported receiving informational support from the healthcare workers. This was in the form of receiving education on the etiology and pathophysiology of ESKD, practical advice on caregiving, dietary requirements, scheduled hospital appointments, and treatment regimen which facilitated adherence to therapeutic instructions. Indeed, inadequate information among family caregivers contributes to feelings of inadequacy in coping with and providing care for a child with ESKD [34, 53]. Thus, assessment of information needs of family caregivers and caregiver-centered educational interventions are crucial to improving their ability to deliver care as well as managing the impact of caregiving on their quality of life. Social support, the capabilities of the family, as well as spiritual beliefs, are considered helpful in adaptation to the situation [13].

### Strength and limitations

This study is the first of its kind to report on the experiences of family caregivers of children with ESKD in Ghana. The researchers were guided by the consolidated criteria for reporting qualitative (COREQ) research in conducting and writing the research report [55]. Guided by the COREQ checklist, we have described the research process, research participants, and interview procedure in adequate detail to help readers make a judgment of the transferability of the findings to other settings. While all participants had children with a confirmed diagnosis of CKD, the data on eGFR was lost owing to certain extenuating circumstances. Like all qualitative studies, the findings cannot be generalized but the study has generated key issues that are transferrable to other similar settings.

## Conclusions

Family caregivers of children with ESKD experience a high burden due to the prolonged and demanding nature of ESKD, greatly affecting their physical, psychological, social, and spiritual well-being. The findings of our study revealed that there is limited funding support for children with ESKD, engendering greater financial challenges for the caregivers and their families. Enhancing the provision of social support, financial support, and health education is instrumental in improving the well-being of family caregivers. The establishment of a support group among the caregivers will be instrumental in the provision of care, especially for newly diagnosed families. Reviewing labor policies to provide paid time off work for civil workers would support sustainable financial support for family caregivers of children with ESKD and their families.

### Electronic supplementary material

Below is the link to the electronic supplementary material.

Supplementary Material 1

Supplementary Material 2

Supplementary Material 3

## Data Availability

The datasets used and/or analyzed during the current study are available from the corresponding author upon reasonable request.
