# Supplementary material for: A qualitative study on the experiences of family caregivers of children with End Stage Kidney Disease (ESKD)
Source: Biopsychosoc Med. 2024 Aug 16;18:17. doi: 10.1186/s13030-024-00314-8 (PMC11328486; doi:10.1186/s13030-024-00314-8)
Supplement: Supplementary file 1 — Supplementary Material 1 [file 13030_2024_314_MOESM1_ESM.docx]

# INTERVIEW GUIDE

Study Title: A qualitative study on the experiences of informal caregivers of children with chronic kidney disease (CKD)

1. Please tell me how you felt after you were informed of your child’s diagnosis
2. Please share with me the event(s) that led to the diagnosis of your child
3. How did you cope with your child’s diagnosis?

Probes

- Personal adjustment
- Family support
- Social support

1. How has the care you are providing for your child affected you physically?

Probes

- Sleep deprivation
- Fatigue
- Stress
- Personal grooming
- Aches/pain
- Loss of appetite

1. How has your child’s disease affected your relationships?

Probes

- Marriage
- Family
- Friends
- Family responsibility
- Role conflict
- Affection/sexual function

1. Please share with me how your child’s condition has affected your work.

Probes

- Employment status
- Attendance to work
- Concentration at work
- What is your financial status

1. Tell me about how your child’s condition has affected your spiritual life

Probes

- The meaning you attribute to your child’s condition
- Hope
- Religiosity

1. Kindly share how you could best be supported to provide care for your child.
