# Supplementary material for: A qualitative study on the experiences of family caregivers of children with End Stage Kidney Disease (ESKD)
Source: Biopsychosoc Med. 2024 Aug 16;18:17. doi: 10.1186/s13030-024-00314-8 (PMC11328486; doi:10.1186/s13030-024-00314-8)
Supplement: Supplementary file 2 — Supplementary Material 2 [file 13030_2024_314_MOESM2_ESM.doc]

**Manuscript:** A qualitative study on the experiences of informal caregivers of children with chronic kidney disease (CKD)

Edward Appiah Boateng, Mabel Baaba Bisiw, Rosemary Agyapomah, Isaac Enyemadze, Joana Kyei-Dompim, Samuel Peprah Kumi, Dorothy Serwaa Boakye

**Consolidated criteria for reporting qualitative studies (COREQ): 32-item checklist**

Developed from:

Tong A, Sainsbury P, Craig J. Consolidated criteria for reporting qualitative research (COREQ): a 32-item checklist for interviews and focus groups. *International Journal for Quality in Health Care*. 2007. Volume 19, Number 6: pp. 349 – 357

| **No. Item** | **Guide questions/description**  **Author response/explanation** | **Reported on Page #** |
| --- | --- | --- |
| **Domain 1: Research team and reﬂexivity** |  | Page |
| *Personal Characteristics* |  |  |
| 1. Interviewer/facilitator | Which author/s conducted the interview or focus group?  MBB | Page 5 |
| 2. Credentials | What were the researcher’s credentials? E.g. PhD, MD  EAB – PhD, RN  MBB – MPhil, RN  RA – MPhil, RN  IE – PhD  JKD – MPhil, RN  SPK – BSc, RN  DSB – PhD Candidate, MPhil, RN | N/A |
| 3. Occupation | What was their occupation at the time of the study?  EAB, JKD, and DSB are registered nurses and university lecturers  MBB – was a registered nurse and a tutor at a nursing and midwifery training college  RA and SPK are registered nurses and tutors of nursing and midwifery training colleges  IE is a tutor in a senior high school | N/A |
| 4. Gender | Was the researcher male or female?  EAB – male  MBB – female  RA – female  IE – male  JKD – female  SPK – male  DSB – female | N/A |
| 5. Experience and training | What experience or training did the researcher have?  EAB, IE, JKD and DSB are experienced researchers with a number of publications  MBB, RA and SPK had taken various courses on research methods | N/A |
| *Relationship with participants* |  |  |
| 6. Relationship established | Was a relationship established prior to study commencement?  After obtaining ethics approval, EAB and MBB visited the pediatric renal unit and explained the purpose, objectives, and process of data collection to the head of the unit | Page 6 |
| 7. Participant knowledge of the interviewer | What did the participants know about the researcher? e.g. personal goals, reasons for doing the research  Data collection was led by MBB who interacted one-on-one with prospective participants to explain the objectives of the study and invited them to participate. | Page 7 |
| 8. Interviewer characteristics | What characteristics were reported about the interviewer/facilitator? e.g. Bias, assumptions, reasons and interests in the research topic  These have been provided. | Page 6 |

| **Domain 2: study design** |  |  |
| --- | --- | --- |
| *Theoretical framework* |  |  |
| 9. Methodological orientation and Theory | What methodological orientation was stated to underpin the study? e.g. grounded theory, discourse analysis, ethnography, phenomenology, content analysis  This study employed a phenomenological approach | Page 6 |
| *Participant selection* |  |  |
| 10. Sampling | How were participants selected? e.g. purposive, convenience, consecutive, snowball  The purposive sampling technique was used to recruit 12 informal caregivers for the study. | Page 6 |
| 11. Method of approach | How were participants approached? e.g. face-to-face, telephone, mail, email  Individual face-to-face interviews were conducted in Twi or English, based on each participant’s choice. | Page 7 |
| 12. Sample size | How many participants were in the study?  A total of 12 informal caregivers, comprising eleven females and one male were recruited for the study. | Page 8 |
| 13. Non-participation | How many people refused to participate or dropped out? Reasons?  All those who were approached agreed to be part of the study | Page 7 |
| *Setting* |  |  |
| 14. Setting of data collection | Where was the data collected? e.g. home, clinic, workplace  All interviews were conducted at a place within the hospital that ensured privacy during the session – mainly in a consulting room. | Page 7  . |
| 15. Presence of non-participants | Was anyone else present besides the participants and researchers?  No third parties were allowed into the interview premises. | Page 7 |
| 16. Description of sample | What are the important characteristics of the sample? e.g. demographic data, date  These have been provided. | Pages 8 and 9 |
| *Data collection* |  |  |
| 17. Interview guide | Were questions, prompts, guides provided by the authors? Was it pilot tested?  Two interviews were conducted to pretest the interview guide that was developed for this study after which minor revisions were made to clarify some questions to elicit in-depth responses from participants.  The interview was initiated with the question: “Please tell me how you felt after you were informed of your child’s diagnosis?” Probes such as “Kindly elaborate further”, or “How do you mean”? were introduced to assist participants in providing in-depth descriptions of their experiences. | Pages 6 and 7 |
| 18. Repeat interviews | Were repeat interviews carried out? If yes, how many?  There were no repeat interviews | Page 7 |
| 19. Audio/visual recording | Did the research use audio or visual recording to collect the data?  The interviews were audio recorded with the consent of participants. | Page 7 |
| 20. Field notes | Were ﬁeld notes made during and/or after the interview or focus group?  Field notes were taken to record observations and mannerisms that were not captured through the audio recording during data collection. | Page 7 |
| 21. Duration | What was the duration of the interviews or focus group?  Each interview session lasted between 25 to 40 minutes | Page 7 |
| 22. Data saturation | Was data saturation discussed?  Yes – The sample size was informed by data saturation where no new themes were derived from the data in line with the objectives of the study as well as the timelines for the completion of the study | Page 6 |
| 23. Transcripts returned | Were transcripts returned to participants for comment and/or correction?  Member checking was also achieved by nine participants confirming that the findings of the study were true reflections of their descriptions. | Page 8 |
| **Domain 3: analysis and ﬁndings** |  |  |
| *Data analysis* |  |  |
| 24. Number of data coders | How many data coders coded the data?  Data analysis was led by MBB, with significant inputs from all authors during regular discussions on the study. | Page 8 |
| 25. Description of the coding tree | Did authors provide a description of the coding tree?  No | N/A |
| 26. Derivation of themes | Were themes identiﬁed in advance or derived from the data?  The pre-existing themes from the adopted model guided the data analysis.  An additional theme titled ‘health education as a coping strategy’ was derived from the data. | Pages 8 |
| 27. Software | What software, if applicable, was used to manage the data?  MS Word software was used to manage and organize the data. | N/A |
| 28. Participant checking | Did participants provide feedback on the ﬁndings?  Yes | Page 8 |
| *Reporting* |  |  |
| 29. Quotations presented | Were participant quotations presented to illustrate the themes/ﬁndings? Was each quotation identiﬁed? e.g. participant number  Yes. Participant numbers were used to identify each quotation. | Pages 8 to 15 |
| 30. Data and ﬁndings consistent | Was there consistency between the data presented and the ﬁndings?  Yes | Pages 8 to 15 |
| 31. Clarity of major themes | Were major themes clearly presented in the ﬁndings?  Yes | Pages 8 to 15 |
| 32. Clarity of minor themes | Is there a description of diverse cases or discussion of minor themes?  Yes, there is a discussion of major and minor themes | Pages 15 to 19 |
