# Supplementary material for: A qualitative study on the experiences of family caregivers of children with End Stage Kidney Disease (ESKD)
Source: Biopsychosoc Med. 2024 Aug 16;18:17. doi: 10.1186/s13030-024-00314-8 (PMC11328486; doi:10.1186/s13030-024-00314-8)
Supplement: Supplementary file 3 — Supplementary Material 3 [file 13030_2024_314_MOESM3_ESM.docx]

Department of Nursing

Kwame Nkrumah University of Science and Technology

PMB

Kumasi – Ghana

7^th^ August 2024

BMC BioPsychoSocial Medicine

BioMed Central

Floor 6, 236 Gray's Inn Road

London

WC1X 8HB

United Kingdom

Dear Editor,

**Revisions to the manuscript titled “A qualitative study on the experiences of family caregivers of children with end stage kidney disease (ESKD)”**

Thank you very much for the opportunity to modify English expressions according to Reviewer #3, the native editor of the journal and to insert a statement suggested by Reviewer #2. All the requested changes have been made to the manuscript.

I am sincerely grateful for all the careful reviews and constructive feedback that have improved the quality of the manuscript.

Thank you.

Yours faithfully,

Edward Appiah Boateng
